# Effects of Delayed Mating on Mating Performance and Reproductive Fitness of the Willow Leaf Beetle (Coleoptera: Chrysomelidae) under Laboratory Conditions

**DOI:** 10.3390/insects12060481

**Published:** 2021-05-21

**Authors:** Lvquan Zhao, Zheng Liu, Yuqun Lin, Shouzhu Liu

**Affiliations:** 1 Co-Innovation Center for Sustainable Forestry in Southern China, College of Forestry, Nanjing Forestry University, Nanjing 210037, China; liuzheng@njfu.edu.cn (Z.L.); qiuying@njfu.edu.cn (Y.L.); 2 School of Agriculture, Liaocheng University, Liaocheng 252059, China; liushouzhu@lcu.edu.cn

**Keywords:** *Plagiodera versicolora*, fecundity, fertility, mating disruption, female longevity

## Abstract

**Simple Summary:**

Mating disruption is one of the most effective methods for pest management. The willow leaf beetle *Plagiodera versicolora* Laicharting is a serious pest of poplar and willow. We studied the effects of delayed mating on the mating performance and reproductive fitness of *P. versicolora*, as part of an effort to control *P. versicolora* using mating disruption. Delayed mating of females and males impacts their mating performance, but has only a limited impact on the females’ reproductive fitness. These results indicate that delayed mating is unlikely to contribute to the success of mating disruption, when applied to control *P. versicolora*.

**Abstract:**

Age at mating is one of the most important factors that affect mating selection, sexual performance, and fecundity. We studied the effects of mating age on the mating performance and reproductive fitness of *Plagiodera versicolora* Laicharting, a serious pest of poplar and willow, by measuring the time from pairing to successful mating, mating duration, fecundity, hatching probability, and female lifespan. Delayed mating of females and males significantly prolonged the time from pairing to successful mating and the mating duration, but had no effect on the duration of the egg-laying period. Delayed mating of females did not significantly affect fecundity or egg hatching, but significantly prolonged the female lifespan. Although delayed mating of males had a significant negative impact on egg hatching, it had no effect on the lifespan or fecundity of females. These results indicate that delayed mating affects the mating performance of *P. versicolora*, although it has a limited effect on reproductive fitness. This suggests that delayed mating is unlikely to contribute to the success of mating disruption, when applied to control *P. versicolora*.

## 1. Introduction

Delayed mating is defined as a condition in which female and/or male mating happens at later stages of life, and it is widespread in nature [1,2]. In insects, delayed mating can be caused by biological and environmental factors, such as mating disruption, trapping, rainfall, unsuitable temperature, and wind. Age at mating affects insects’ mating selection, sexual performance, and fecundity [3,4,5]. The physiological status of adults changes over time; therefore, mating between young males and females can increase their reproductive fitness [6]. For example, males preferentially select young females for mating, and this provides a reproductive advantage of producing relatively more offspring [7,8]. Females mating with young males can obtain a higher breeding value and fewer deleterious mutations [9,10].

The effects of delayed mating on fecundity, egg hatching, and lifespan have been reported in Coleoptera, Lepidoptera, Diptera, and Hemiptera, and show that fecundity decreases or remains constant due to delay in mating, but does not increase [11]. For example, delayed mating of the Coleoptera *Anomala orientalis* (Waterhouse) has a significant negative effect on female reproductive output [11]. In Lepidoptera, many studies have demonstrated that delayed mating has a negative impact on fecundity and egg hatching. For example, delayed mating reduces the mating success rate of *Phauda flammans* (Walker), and reduces the fecundity and egg hatching of mated females [12]. Other Lepidoptera such as *Cnaphalocrocis medinalis* (Guenée) [13], *Ectropis obliqua* Prout [14], and *Chilo partellus* (Swinhoe) [15] have exhibited similar effects. However, delayed mating of the Dipteran *Lycoriella ingenua* (Dufour) did not affect female fecundity or egg hatching [16]. Delayed mating of the Hemipteran mealybug, *Planococcus ficus* (signoret), had no effect on fecundity, while delayed mating of males significantly improved egg hatching [17]. These results indicate that the effects of delayed mating on fecundity and egg hatching can vary among species.

The willow leaf beetle *Plagiodera versicolora* Laicharting (Coleoptera: *Chrysomelidae*) is widely distributed in Asia, Europe, and North Africa. Larvae and adults of *P. versicolora* feed on poplar and willow leaves and twigs, leaving only the midrib and a network of veins [18]. The damage done by this pest can seriously affect the growth of seedlings and saplings. The conventional control strategy of using chemical insecticides can efficiently reduce the population density, but their long-term application inevitably leads to undesirable and hazardous side effects for the environment, plants, and human life [19]. In addition, the *Bacillis thuringiensis* (Bt) toxin and RNA interference (RNAi) technology are being directed towards developing new methods for *P. versicolora* control [18,20,21,22]. However, the effects of delayed mating on mating performance and reproductive fitness are not known. We studied the effects of delayed mating on the mating performance and reproductive fitness of *P. versicolora* in order to understand the potential of mating disruption, such as by the application of sex pheromones, for managing *P. versicolora*. We evaluated the effects of delayed mating on the time from pairing to successful mating, mating duration, female fecundity and egg hatching, female lifespan, and the egg laying patterns of *P. versicolora*.

## 2. Materials and Methods

### 2.1. Insect Rearing

Larvae of *P. versicolora* were collected from their host plant, *Salix babylonica*, in April 2019 on the campus of Nanjing Forestry University, Nanjing City, Jiangsu Province. The larvae were taken to the laboratory and reared in plastic containers (5 cm diameter × 4 cm high). The leaves were replaced daily until the larvae pupated. The pupae were moved to a new container for adult emergence. Newly emerged adults were fed with fresh *S. babylonica* leaves in groups within plastic containers. The adults oviposited on the willow leaves, and the eggs laid by the adults were collected daily and transferred to a petri dish with moist filter paper on the bottom. Two generations were reared as described above. The newly hatched larvae of the third generation were transferred to a new container and reared separately, and the adults, after emergence, were used for experiments. All experiments were carried out in an incubator at 25 ± 1 °C and with an L16:D8 photoperiod.

### 2.2. Experimental Design

The adults were reared separately after emergence, and their fresh body weight was measured using an electronic balance (AL104 Mettler Toledo; Mettler Toledo, d = 0.0001 g) on the second day after adult emergence. We determined the sex of each adult by their body weight, and the adults were considered to be females if their body weight was more than 6.5 mg; conversely, the adults were considered to be males if their body weight was less than 6.5 mg. We validated this method of sexing adults by mating behavior, with a success rate of 95%. The lifespan of unmated females is about 50 d, and the pre-oviposition period of females is about 7 d [23]. Therefore, we assume that female and male adults may be encountered at various ages under field conditions. The experiment was divided into two groups: the first group included 7-d-old virgin females paired with virgin males at 7 d, 14 d, or 21 d after male emergence. The second group included 7-d-old virgin males paired with virgin females at 7 d, 14 d, or 21 d after female emergence. Twenty-eight females and males at seven days and thirty females and males at fourteen days and twenty-one days were used in the above experiments. When pairing, the virgin females and males, which were reared individually, were moved to a new plastic container, and the male would actively approach and touch the female with its antennae. It would then crawl onto the female’s back and insert its aedeagus into the female genitalia, and after mating would pull out its aedeagus. We scored a successful mating as one in which the male aedeagus remained in the female for at least 20 min. All of the females and males mated successfully within 20 min. Two matings are needed in order to fertilize all of the eggs of female *P. versicolora* [23]. To compare the effects of delayed mating on mating performance and reproductive fitness, we permitted females and males to mate only once. We recorded the time from pairing to successful mating (the time from moving the female and male to the container to the male inserting his genitalia into the female genitalia) and the mating duration (the time from the male inserting his genitalia into the female genitalia to the retraction of his genitalia) in the above mating experiment.

### 2.3. Measurements of Reproductive Fitness

We used three quantitative indicators to compare female reproductive fitness after mating. These were fecundity, egg hatching, and female lifespan. After the females and males mated successfully, the females were reared separately until they died, according to the method described in the insect breeding section, and the males were discarded. The females laid their eggs on willow leaves in egg clutches, and the number of eggs per clutch varied from 1 to 37 (average = 15) [24]. Once the females began to lay eggs, the egg clutches of each mated female were transferred, along with the willow leaves, to a petri dish with moist filter paper for egg counts and egg hatching per clutch over her life span. We also checked and recorded the egg-laying duration (the time from first oviposition to last oviposition) and female survival in the mating schedule given above.

### 2.4. Statistical Analysis

The experimental data were analyzed in SPSS 22.0 (IBM Inc., New York, NY, USA). We used one-way analysis of variance (ANOVA) for the time from pairing to successful mating, mating duration, the duration of the egg-laying period, the number of egg clutches, the number of eggs per clutch, and egg hatching. Comparison of female survival among treatments was performed using Kaplan–Meier estimation and log-rank tests. Covariance analysis (ANCOVA) was performed on fecundity using female body weight as a covariate. When the F value was significant (*p* < 0.05), we used Fisher’s least significant difference test for pairwise comparisons. The eggs of some females were lost during the study, so only 28 females at 14 d and 21 d, 28 females at 14 d, and 27 females at 21 d were used to investigate the effects of delayed female mating and delayed male mating on fecundity and egg hatching, respectively.

## 3. Results

### 3.1. Time from Pairing to Successful Mating and Mating Duration

**Figure 1:**
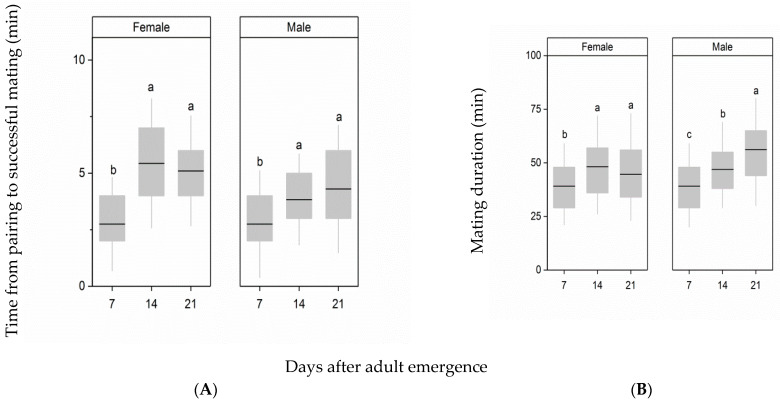
Effects of delayed mating of females and males on the time from pairing to successful mating (**A**) and on mating duration (**B**). Different letters indicate significant differences from one another at *p* < 0.05. The top and bottom of each box represent the upper and lower quartiles, respectively; the horizontal line represents the median; the vertical lines extend to the minimum and maximum values within 1.5 times the interquartile range.

Delayed mating of females and males has a significant effect on the time from pairing to successful mating (female: F_2,85_ = 19.05, *p* < 0.001; male: F_2,85_ = 5.52, *p* = 0.0056, Figure 1A). When the age of females at mating was delayed from 7 d to 14 d or 21 d after emergence, the time from pairing to successful mating was prolonged, and it was significantly longer than the time from pairing to successful mating when females at 7 d after emergence were paired with males (*p* < 0.001 in all cases). Similarly, the time from pairing to successful mating when the males at 14 d or 21 d after emergence were paired with females was also significantly longer than the time from pairing to successful mating when the males at 7 d after emergence were paired with females (day 14: *p* = 0.033; day 21: *p* = 0.0013).

Delayed mating of females and males had a significant effect on mating duration (female: F_2,85_ = 4.48, *p* = 0.0031; male: F_2,85_ = 9.83, *p* < 0.001, Figure 1B). The mating duration of females at 14 d or 21 d after emergence was similar (*p* = 0.061), but they were significantly longer than that of females at 7 d after emergence (day 14: *p* = 0.0089; day 21: *p* = 0.024). Similarly, as the age of males was increased prior to mating, the mating duration was also significantly extended (day 14: *p* = 0.019; day 21: *p* < 0.001).

### 3.2. Duration of the Egg-Laying Period and Female Lifespan

**Figure 2:**
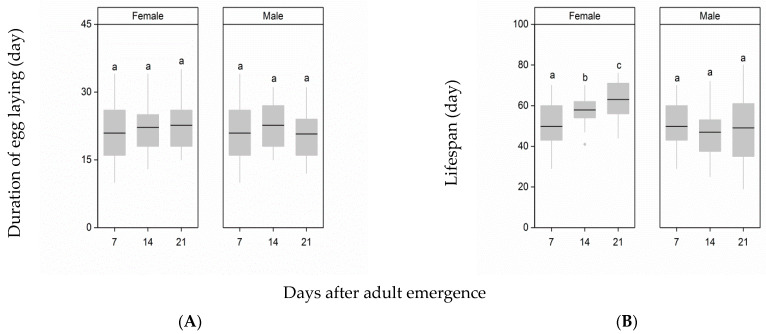
Effects of delayed mating of females and males on the female post-mating duration of the egg-laying period (**A**) and on life span (**B**). Different letters indicate significant differences from one another at *p* < 0.05. The top and bottom of each box represent the upper and lower quartiles, respectively; the horizontal line represents the median; the vertical lines extend to the minimum and maximum values within 1.5 times the interquartile range.

Delayed mating of females and males did not significantly influence the duration of the egg-laying period (Female: F_2,85_ = 0.73, *p* = 0.48; male: F_2,85_ = 0.96, *p* = 0.38, Figure 2A). Delayed mating of females had a significant impact on female lifespan, but delayed mating of males had no effect on the lifespan of females (female: χ^2^ = 8.54, *p* < 0.0001; male: χ^2^ = 1.56, *p* = 0.84, Figure 2B). When the age of females at mating was delayed from 7 d to 14 d or 21 d after emergence, the lifespan of females was also extended, and there were significant differences between all treatments (*p* < 0.05 in all cases).

### 3.3. Fecundity and Egg Hatching Rate

**Figure 3:**
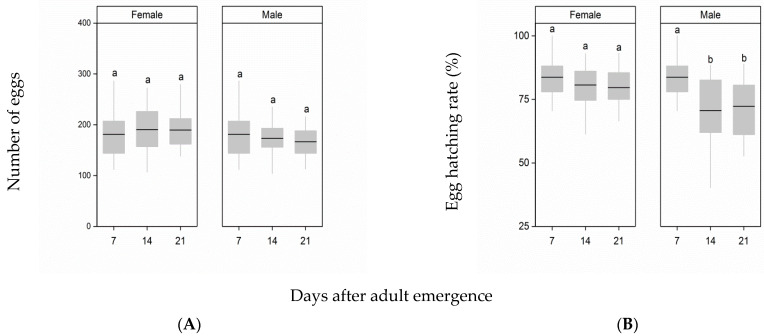
Effects of delayed mating of females and males on female post-mating fecundity (**A**) and on the egg hatching rate (**B**). Different letters indicate significant differences from one another at *p* < 0.05. The top and bottom of each box represent the upper and lower quartiles, respectively; the horizontal line represents the median; the vertical lines extend to the minimum and maximum values within 1.5 times the interquartile range.

Delayed mating of females and males did not significantly influence fecundity (female: F_2,81_ = 0.77, *p* = 0.46; male: F_2,81_ = 0.85, *p* = 0.43, Figure 3A). Although delayed female mating had no effect on the egg hatching rate, male delayed mating had a significant negative effect on the egg hatching rate (female: F_2,80_ = 1.84, *p* = 0.16; male: F_2,80_ = 3.28, *p* = 0.042, Figure 3B). When the age of males mated to females increased from 7 d to 14 d or 21 d after emergence, the egg hatching rate decreased, and it was significantly lower than the egg hatching rate of females mated to males at 7 d after emergence (day 14: *p* = 0.017; day 21: *p* = 0.0026).

### 3.4. Number of Egg Clutches per Female and Number of Eggs per Clutch

**Figure 4:**
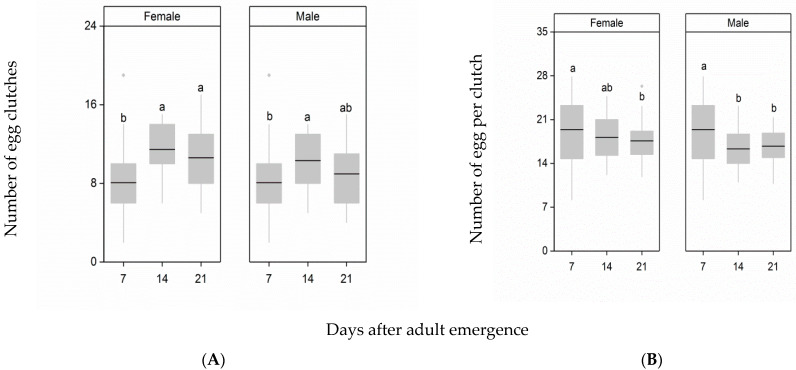
Effects of delayed mating of females and males on the female post-mating number of egg clutches per female (**A**) and on the number of eggs per clutch (**B**). Different letters indicate significant differences from one another at *p* < 0.05. The top and bottom of each box represent the upper and lower quartiles, respectively; the horizontal line represents the median; the vertical lines extend to the minimum and maximum values within 1.5 times the interquartile range.

To clarify the effect of delayed mating of females and males on female egg-laying patterns, we recorded the number of egg clutches per female and the number of eggs per clutch. In contrast to the finding that delayed mating had no significant effect on fecundity, delayed mating significantly increased the number of egg clutches (female: F_2,87_ = 8.28, *p* < 0.001; male: F_2,87_ = 2.92, *p* = 0.042, Figure 4A). When the mating age of females was delayed from 7 d to 14 d or 21 d after emergence, the number of egg clutches increased, and was significantly greater than the number of egg clutches of females mating at 7 d after emergence (day 14: *p* < 0.001; day 21: *p* = 0.011). The number of egg clutches produced by females mating with males at 7 d and 21 d after adult emergence was similar (*p* = 0.026), but the number of egg clutches produced by females mating at 7 d after adult emergence was significantly smaller than that of females mating with males at 14 d after emergence (*p* = 0.17).

Delayed mating of females and males significantly reduced the number of eggs per clutch (female: F_2,87_ = 3.24, *p* = 0.034; male: F_2,87_ = 6.81, *p* = 0.0018, Figure 4B). Although the number of eggs per clutch after mating between males and females mated at 7 d after emergence was similar to that of females at 14 d after emergence (*p* = 0.13), it was significantly larger than the number of eggs per clutch after mating between males and females mated at 21 d after emergence (*p* = 0.034). The number of eggs per clutch produced by females mating with males at 7 d after emergence was significantly greater than that of females mating with males at 14 d and 21 d after emergence (day 14: *p* = 0.0029; day 21: *p* = 0.011).

## 4. Discussion

The effect of delayed mating on mating duration has been reported in other species. For example, delayed mating significantly increased the duration of mating between males and females of *Spodoptera litura* Fabricius [25]. *Plutella xylostella* (L.) showed similar results [1]. Our results are consistent with these findings. Delayed mating significantly prolonged the mating duration of females and males of *P. versicolora*. There is often a significant correlation between female fertilization rate and mating duration [26]. One mating cannot fertilize all of the eggs of female *P. versicolora* [23]. Therefore, when the female and male delay mating, the female may lay a similar or greater number of eggs by extending the mating duration and maximizing the reproductive output.

Zhang et al. [27] conducted a meta-analysis on the effects of delayed mating on the reproductive fitness of moths, and showed that delayed mating had a significant negative impact on fecundity and egg hatching. However, the results of this study showed that delayed mating had no significant effect on fecundity. Successive mating or excessive frequency of mating can shorten the oviposition time of female *P. versicolora*, which can decrease their fecundity [23]. However, an increase in adult density significantly prolongs the time in which for females to lay eggs, thereby promoting an increase in female fecundity [28]. Therefore, we demonstrated that delayed mating has no effect on fecundity, and suggest that this is related to the finding that delayed mating had no effect on the duration of the egg-laying period. Wenninger and Averill [11] showed that delayed mating in *A. orientalis* decreased fecundity and also significantly reduced egg-laying time. In *P. ficus*, the delayed mating of females did not affect oviposition time, and did not affect the number of eggs laid [17]. The above results confirm our speculation. In theory, if females have a fixed lifespan, delayed mating reduces the time available to oviposit, and negatively affects their fecundity. For *P. versicolora*, delayed-mating females attain an oviposition time similar to that of non-delayed-mating females by extending their lifespan under laboratory conditions. This ensures that their fecundity is not affected by delayed mating. A similar result was also reported in *P. ficus*, where delayed mating had no effect on fecundity because the delayed-mating females significantly prolonged their longevity [29]. For the males that delayed mating, the lifespan of their mated female partners did not change significantly, so there was no effect on their oviposition time or fecundity.

In the present study, females mated at older ages lived longer compared to females mated earlier; similar results have been recorded in *Phenacoccus solenopsis* and other species [2]. Increased longevity in delayed-mating females is considered to come from the lesser energy utilization associated with reproduction, and from the reabsorption of food nutrients from exiting ova [11,30]. This suggests that *P. versicolora* can alter their energy utilization pathways in order to attain a good adaptive value to maintain their fitness, so that females mated at older ages can benefit from future opportunities for copulation and progeny production than females mated earlier [31].

Delayed mating of females had no effect on egg hatching, while delayed mating of males significantly reduced egg hatching. A possible explanation is that delayed mating may reduce the quality of male sperm, and thereby reduce egg hatching. Sperm quality can decrease with male age, which can lead to decreased ability to inseminate females [32,33]. We found that males can mate normally at 7 d after emergence. Therefore, when males at 14 d or 21 d post-emergence mate with females, it is possible that their sperm quality has declined. However, one mating cannot fertilize all of the eggs of female *P. versicolora* [23]. Therefore, when males delay mating, decreased sperm quality will reduce their ability to inseminate females and reduce egg hatching. When delayed-mating females mated with males at 7 d after emergence, the egg hatching did not change significantly, which supports our hypothesis. Another possible explanation is that delayed mating could decrease the number of spermatophores transferred by males, and thus decrease egg production [13]. A similar negative relationship between male delay in mating and fecundity has been reported in *P. xylostella* and *P. solenopsis* [1,2].

The egg-laying pattern of *P. versicolora* is affected by mating frequency, mating pattern, and adult density [23,28]. This study showed that delayed mating can also change the female egg-laying pattern. Although delayed mating reduced the number of eggs per clutch, delayed mating significantly increased the number of female egg clutches. The changes in oviposition patterns may be an adaptation of *P. versicolora* to delayed mating. Under laboratory conditions, the lifespan of females after one mating is about 50 days [23], while under natural environmental conditions, the lifespan of females is probably shorter due to natural enemies, extreme temperatures, and food quality. As the age of females at mating increases, the time available for females to lay eggs will decrease. Therefore, when the delayed-mating females start to oviposit, increasing the number of egg clutches can effectively increase the number of offspring, maximizing the reproductive output. The effect of males’ age at mating on female fecundity remains to be studied.

## 5. Conclusions

This study showed that delayed mating of *P. versicolora* adults prolonged the time from pairing to successful mating, as well as the duration of mating, but it had no significant effect on the duration of the egg-laying period. Delayed mating of females did not significantly affect fecundity or egg hatching. Delayed mating of males reduced egg hatching, but did not significantly affect female fecundity. These results may indicate that the efficacy of mating disruption should not count on reduced fecundity due to mating at older ages, as has been suggested for other pest species [16].

## Data Availability

The data presented in this study are available on request from the corresponding author.
